# The relation between perception of vulnerability, stress, depression and anxiety in public health threats

**DOI:** 10.1192/j.eurpsy.2025.2445

**Published:** 2025-08-26

**Authors:** C. de Sousa, J. Ferreira, S. Tukaiev

**Affiliations:** 1 Higher School of Health Atlântica, Barcarena; 2 Center for Research in Education and Psychology (CIEP-UÉ), Evora; 3 Atlântica - University Institute, Barcarena; 4 Faculty of Medicine, University of Coimbra, coimbra, Portugal; 5 National University of Ukraine on Physical Education and Sport, Kyiv, Ukraine

## Abstract

**Introduction:**

Several authors have demonstrated that COVID-19 pandemic was characterized by feelings of fear, anxiety and insecurity (Wang *et al*., Int JERPH 2020; 17, 1729) Emotions and affective states as nervous, apprehension, despair, preoccupation was also studied (de Sousa *et al*., *PSYCH* 2024; *6*(1), 163-176). Therefore, affective but also cognitive factors were important to the perception of the pandemic threat and its impact. In accordance, it seems important to understand if the perception of each one vulnerability to COVID-19 is related with the development of high levels of stress, depression and anxiety symptoms.

**Objectives:**

The main goal of this study was to evaluate the relation between the perception of vulnerability, stress, depression and anxiety in public health threats, as COVID 19.

**Methods:**

This study followed a cross-sectional design and its sample consisted of 600 participants, distributed between two countries (N _Brasil_=300; N _Portugal_=300). The instrument used to assess the three dimensions: stress, anxiety, and depression, with seven items each were the Depression Anxiety Stress Scale 21-item version (DASS-21) (Lovibond & Lovibond; BHV, 1995; 33, 335-43 ) presented in a four-point response scale (0-Not applied to me; 3- Applied to me most of the time). To assess the perception of vulnerability to COVID-19 a 5 item Likert Scale were used (1-Not vulnerable; 5-Extremely vulnerable). The protocol was developed online, presenting the objectives of the study, and ensuring the anonymity and confidentiality of data. A non-probabilistic sampling technique of convenience and snowball were used. The data was collected in 2023 and referred to COVID-19.

**Results:**

The results suggest that perception of vulnerability to COVID-19 are very similar in both countries (*M*_Brazil_= 3.20, *DP*_Brazil_= .98; *M*_Portugal_= 3.13, *DP*_Portugal_=.91). The values in Brazil of stress (*M*=7.40), depression (*M*=5.73) and anxiety (*M*=4.01) are higher compared to Portugal, with the following values of stress (*M*=4.07), depression (*M*=2.62) and anxiety (*M*=1.6). The perception of vulnerability is related with the three dimensions studied (stress, anxiety and depression) in both countries, with the following values in Brasil: Stress (*r*=.341; *p*< .01); depression (*r*=.270; *p*<.01) and anxiety (*r*=.316; *p*<.01). In Portugal the values of correlation were: Stress (*r*=.284; *p*< .01); depression (*r*=.252; *p*<.01) and anxiety (*r*=.350; *p*<.01). These results emphasized that relation between the perception of vulnerability and anxiety presented higher levels in both countries.

**Conclusions:**

In conclusion, our study have shown significative relations between the perception of vulnerability to COVID-19 with levels of stress, depression and anxiety in the countries studied. Therefore, our data emphasized the relevance to study the perception of vulnerability in public health events to better manage and prevent psychopathological symptoms.

**Disclosure of Interest:**

None Declared

